# Influence of hiatal hernia and male sex on the relationship between alcohol intake and occurrence of Barrett’s esophagus

**DOI:** 10.1371/journal.pone.0192951

**Published:** 2018-02-15

**Authors:** Atsuhiro Masuda, Tsuyoshi Fujita, Manabu Murakami, Yukinao Yamazaki, Masao Kobayashi, Shuichi Terao, Tsuyoshi Sanuki, Akihiko Okada, Masayasu Adachi, Hideyuki Shiomi, Yoshifumi Arisaka, Hiromu Kutsumi, Eiji Umegaki, Takeshi Azuma

**Affiliations:** 1 Division of Gastroenterology, Department of Internal Medicine, Kobe University Graduate School of Medicine, Hyogo, Japan; 2 Department of Gastroenterology, Yodogawa Christian Hospital, Osaka, Japan; 3 Department of Gastroenterology, Fukui Red Cross Hospital, Fukui, Japan; 4 Department of Health Care, Kyoto Second Red Cross Hospital, Kyoto, Japan; 5 Department of Gastroenterology, Kakogawa Central City Hospital, Hyogo, Japan; 6 Department of Gastroenterology, Kita-Harima Medical Center, Hyogo, Japan; 7 Department of Gastroenterology, Saiseikai Nakatsu Hospital, Osaka, Japan; 8 Hotel Okura Kobe Clinic, Hyogo, Japan; 9 Clinical Research and Medical Innovation Center, Shiga University Medical Science, Shiga, Japan; Peter MacCallum Cancer Centre, AUSTRALIA

## Abstract

**Background:**

The association of alcohol intake with the incidence of Barrett’s esophagus (BE) has been inconsistent. Although hiatal hernia and male sex are well-known risk factors of BE, its effect on the association of alcohol intake with the incidence of BE remains unknown.

**Aim:**

To investigate whether the influence of alcohol intake on the occurrence of BE might differ depending on male sex and presence of hiatal hernia.

**Methods:**

We utilized a database of 8031 patients that underwent upper endoscopy for health screening in a prospective, multicenter, cohort study (the Upper Gastro Intestinal Disease study). The incidence of endoscopic columnar-lined esophagus (eCLE; endoscopically diagnosed BE) was the outcome variable. Multivariable logistic regression analysis was conducted to assess the association between alcohol intake and eCLE stratified by male sex and hiatal hernia, adjusting for clinical features and other potential confounders.

**Results:**

Alcohol intake (≥20 g/day) showed a marginally significant association with the incidence of eCLE in participants without hiatal hernia (0 vs. ≥20 g/day; odds ratio [OR], 1.62; 95% confidence interval [CI], 0.92–2.85, *P* = 0.09) but not in participants with hiatal hernia (0 vs. ≥20/day; OR, 0.99; 95% CI, 0.59–1.65; *P* = 0.95). Furthermore, alcohol intake (≥20 g/day) was significantly associated with the incidence of eCLE in male participants without hiatal hernia (0 vs. ≥20 g/day; OR, 1.98; 95% CI, 1.04–4.03; *P* = 0.04) but not in female participants without hiatal hernia (0 vs. ≥20 g/day; OR, 0.47; 95% CI, 0.03–2.37; *P* = 0.42).

**Conclusions:**

The effect of alcohol intake on the incidence of eCLE might be associated with hiatal hernia status and male sex.

## Introduction

Barrett’s esophagus (BE) is a strong risk factor for the development of esophageal adenocarcinoma [1–4]. Some meta-analyses have shown that the pooled annual incidence of esophageal adenocarcinoma of BE was 0.19–0.33% [5, 6]. BE has malignant potential, and the determination of its epidemiology is important for preventing and screening for BE-derived esophageal adenocarcinoma.

The relationship of alcohol consumption with occurrence of BE remains controversial. Alcohol consumption causes a reduction in lower esophageal sphincter (LES) pressure and leads to gastroesophageal reflux disease (GERD) symptoms, which may induce BE [7]. Some reports have shown a significant association of alcohol consumption with occurrence of BE [8, 9], but other reports have not [10, 11]. It is important to determine the specific patient characteristics that have a significant relationship with alcohol consumption and occurrence of BE.

Evidence suggested that hiatal hernia is one of the major causes of GERD and BE [12, 13]. In hiatal hernia, the LES pressure is consistently low or weak. Hiatal hernia does not have a normally functioning LES or normal regulation of LES pressure [14]. Meanwhile, male sex is also strongly associated with the occurrence of BE [15, 16]. The anti-inflammatory action of estrogen and esophageal epithelial resistance against reflux of gastric acid was reported as its pathogenesis [17, 18]. Therefore, we investigated whether the effect of alcohol consumption might be associated with hiatal hernia and male sex.

## Materials and methods

### Study subjects

We utilized the database of a prospective, multicenter, cohort study (the Upper Gastro Intestinal Disease [UGID] study) of 8891 subjects enrolled in 2013–2014 who underwent upper endoscopy for health screening at seven facilities. Based on the availability of data on endoscopic columnar-lined esophagus (eCLE; endoscopically diagnosed BE), hiatal hernia, and alcohol consumption, a total of 8031 cases were included. Participants with post-total gastrectomy and those who use proton pump inhibitor and/or histamine H2-receptor antagonist were excluded from this study.

### Prospective questionnaire and endoscopic findings

We collected the data from a prospective questionnaire (S1 File) including age, height, body weight, sex (male, female), current smoking (presence, absence), the current average daily alcohol consumption (0, <20, or ≥20 g/day; cutoff value of 20 g/day determined according to previous Japanese epidemiological studies) [19, 20], and heartburn and/or acid regurgitation (at any frequency in 3 months; presence, absence). Endoscopic findings were examined by experienced endoscopists in each institution, including eCLE (<10 mm, ≥10 mm), erosive esophagitis (presence, absence), hiatal hernia (presence, absence), and atrophic gastritis (presence, absence). Non-erosive reflux disease (NERD; presence, absence) was diagnosed when a subject responded that heartburn and/or acid regurgitation was present at a frequency of at least once per week in the absence of erosive esophagitis [21]. The presence of eCLE was defined as >10-mm length of columnar-lined epithelium on upper endoscopy. Endoscopic columnar-lined epithelium was diagnosed using the palisade vessels as landmark for the esophagogastric junction. An eCLE measuring less than 10 mm, reported as an “ultrashort segment” of eCLE [22], was not considered as the presence of eCLE in this study because its diagnostic criteria have been vague and there might be a high degree of inter-observer variation. Erosive esophagitis was defined by the Los Angeles classification (A-D) [23]. Presence of hiatal hernia was defined as the proximal dislocation of the gastroesophageal junction (GEJ) >2 cm above the diaphragmatic indentation. Severity of hiatal hernia was categorized by the length of the proximal dislocation of GEJ, with 2–4 cm considered as mild and >4 cm as severe. Atrophic gastritis was endoscopically diagnosed, and the endoscopic extent of atrophic mucosa was graded according to the Kimura-Takemoto classification from C-1 to O-3 [24]. Subjects with atrophic mucosa graded as C-2, C-3, O-1, O-2, and O-3 were described as positive for atrophic gastritis. This study was conducted in accordance with the Declaration of Helsinki and its amendments (UMIN-CTR ID: 000022504). The study protocol was approved by the Ethics Committee of each institution (the ethics committee of Yodogawa Christian Hospital, Fukui Red Cross Hospital, Kyoto Second Red Cross Hospital, Kakogawa Central City Hospital, Kita-Harima Medical Center, Saiseikai Nakatsu Hospital, Hotel Okura Kobe Clinic). Written informed consent was obtained from all study participants. All authors had access to the study data and had reviewed and approved the final manuscript.

### Statistical analysis

All statistical analyses were conducted using JMP version 11 (SAS Institute, Cary, NC, USA), and all *P* values were two-sided. First, to identify the candidate risk factors, we conducted multivariable binary logistic regression analysis to assess the risk factors for the occurrence of eCLE. The binary categorical variable of eCLE (<10 mm, ≥10 mm) was used as outcome variables. Multivariable binary logistic regression analysis was performed to adjust for potential confounders. The multivariable model initially included age, body mass index, sex, current smoking, alcohol consumption, presence of heartburn or acid regurgitation, presence of erosive esophagitis, presence of hiatal hernia, presence of atrophic gastritis, and presence of NERD. A backward stepwise elimination with a threshold of *P* = 0.05 was used to select the variables for the final models. Then, we conducted a binary logistic model to assess the association between alcohol consumption and hiatal hernia status in relation to the occurrence of eCLE. Furthermore, we conducted a binary logistic model to assess the association between alcohol consumption and status of hiatal hernia in relation to the occurrence of eCLE in male and female participants. We also conducted a multivariate binary logistic regression analysis to assess the risk factors for the occurrence of eCLE stratified by sex. To assess associations between categorical data, the χ^2^ test (or Fisher’s exact test if appropriate) was performed. To compare mean age and body mass index, a *t*-test or analysis of variance, assuming equal variances, was performed. In all analyses, *P*<0.05 indicates statistical significance.

## Results

### Logistic regression analysis of risk factors for eCLE in the UGID study

In this study, 174 cases of eCLE were observed (Table 1). We conducted a multivariable logistic regression analysis to identify the risk factors for eCLE in all 8031 participants (Table 2). Results showed that the independent risk factors of eCLE were presence of hiatal hernia (odds ratio [OR] = 2.89, 95% confidence interval [CI] = 2.12–3.96, *P*<0.0001), presence of erosive esophagitis (OR = 2.06, 95% CI = 1.48–2.86, *P*<0.0001), age (10-year increments, OR = 1.44, 95% CI = 1.24–1.68, *P*<0.0001), and alcohol consumption (≥20 g/day, OR = 1.58, 95% CI = 1.15–2.16, *P* = 0.005).

**Table 1:** Characteristics of participants in UGID study.

	Total number
All patients	8031
Mean age ± SD (years)	52.6 ± 10.1
Sex	
Men	5014 (62.4%)
Women	3017 (37.6%)
Body Mass Index (kg/m^2^)	
≥ 25	1859 (23.1%)
< 25	6172 (76.9%)
Current smoking	
Presence	1331 (16.6%)
Absence	6700 (83.4%)
Alcohol consumption	
≥ 20g/day	2144 (26.7%)
<20 g/day	2664 (33.2%)
None	3223 (40.1%)
Heartburn or acid regurgitation	
Presence	3384 (42.1%)
Absence	4647 (57.9%)
Endoscopic columnar-lined esophagus	
≥30 mm	17 (0.2%)
≥10 to <30 mm	157 (2.0%)
<10 mm	7857 (97.8%)
Erosive esophagitis	
Presence	1350 (16.8%)
Absence	6681 (83.2%)
Hiatal Hernia	
Presence	2307 (28.7%)
Absence	5724 (71.3%)
Atrophic gastritis	
Presence	3147 (39.2%)
Absence	4884 (60.8%)
NERD	
Presence	389 (4.8%)
Absence	7642 (95.2%)

**Table 2:** Logistic regression analysis of risk factors for endoscopic columnar-lined esophagus (N = 8031).

	OR (95%CI)	*P* value
Univariable analysis		
Hiatal hernia (presence)	3.37 (2.50–4.59)	<0.0001
Erosive esophagitis (presence)	2.82 (2.04–3.85)	<0.0001
Sex (male)	2.18 (1.54–3.18)	<0.0001
Alcohol consumption (≥20 g/day)	1.92 (1.41–2.61)	<0.0001
Age (10-year increments)	1.42 (1.23–1.64)	<0.0001
Heartburn or acid regurgitation (presence)	1.52 (1.12–2.06)	0.006
Current smoking (presence)	1.41 (0.97–2.02)	0.069
BMI (≥25 kg/m^2^)	1.35 (0.96–1.87)	0.085
NERD (presence)	1.20 (0.59–2.18)	0.59
Atrophic gastritis (presence)	1.06 (0.78–1.44)	0.73
Multivariable analysis*		
Hiatal hernia (presence)	2.89 (2.12–3.96)	<0.0001
Erosive esophagitis (presence)	2.06 (1.48–2.86)	<0.0001
Age (10-year increments)	1.44 (1.24–1.68)	<0.0001
Alcohol consumption (≥20 g/day)	1.58 (1.15–2.16)	0.005

Presence of endoscopic columnar-lined esophagus was defined as >10-mm length of columnar-lined esophagus on upper endoscopy.

The risk of endoscopic columnar-lined esophagus was evaluated by age, sex, BMI, current smoking, alcohol consumption, presence of heartburn symptom or acid regurgitation, presence of erosive esophagitis, presence of hiatal hernia, presence of atrophic gastritis, and presence of NERD.

*The odds ratio was adjusted for age, sex, BMI, current smoking, alcohol consumption, presence of heartburn or acid regurgitation, presence of erosive esophagitis, presence of hiatal hernia, presence of atrophic gastritis, and presence of NERD.

BMI, body mass index; CI, confidence interval; NERD, non-erosive reflex disease; OR, odds ratio.

### Logistic regression analysis to assess the association between alcohol consumption and the occurrence of eCLE, stratified by hiatal hernia status

To assess the association in the occurrence of eCLE between alcohol intake and hiatal hernia status, we conducted a binary logistic regression analysis (Table 3). To evaluate the effect of a low alcohol consumption, we used three categories of alcohol intake: 0, <20, and ≥20 g/day. Alcohol intake (≥20 g/day) was marginally significantly associated with the incidence of eCLE in participants without hiatal hernia (0 vs. ≥20 g/day; OR, 1.62; 95% CI, 0.92–2.85; *P* = 0.09) but not in participants with hiatal hernia (0 vs. ≥ 20 g/day; OR, 0.99; 95% CI, 0.59–1.65; *P* = 0.95). In addition, there was no difference in its relationship with the degree of hiatal hernia (length of the proximal dislocation of the GEJ above the diaphragmatic indentation, 2–4 cm vs. >4 cm; S1 Table).

**Table 3:** Logistic regression analysis to assess the association between alcohol consumption and the occurrence of endoscopic columnar-lined esophagus stratified by hiatal hernia status.

				Endoscopic columnar-lined esophagus (Outcome variable†)	
		No. of	No. of	Univariable	Multivariable
		cases	eCLE	OR (95% CI)	OR (95% CI)*
Hiatal hernia (+)	Total	2307	99 (4.3%)		
Alcohol consumption	None	869	35 (4.0%)	1 (reference)	1 (reference)
	< 20g/day	704	27 (3.8%)	0.95 (0.57–1.58)	0.94 (0.55–1.64)
	≥ 20g/day	697	37 (5.0%)	1.26 (0.78–2.04)	0.99 (0.59–1.65)
	*P* value for 0 vs. <20g/day			0.84	0.81
	*P* value for 0 vs. >20g/day			0.33	0.95
Hiatal hernia (-)	Total	5724	75 (1.3%)		
Alcohol consumption	None	2325	29 (1.2%)	1 (reference)	1 (reference)
	< 20g/day	1948	12 (0.6%)	0.49 (0.24–0.95)	0.46 (0.22–0.90)
	≥ 20g/day	1376	34 (2.4%)	1.98 (1.20–3.28)	1.62 (0.92–2.85)
	*P* value for 0 vs. <20g/day			0.03	0.03
	*P* value for 0 vs. >20g/day			0.007	0.09

†Presence of endoscopic columnar-lined esophagus was defined as >10-mm length of columnar-lined esophagus on upper endoscopy.

*Odds ratio was adjusted for age, sex, body mass index, current smoking, presence of heartburn or acid regurgitation, presence of erosive esophagitis, presence of atrophic gastritis, and presence of non-erosive reflex disease.

CI, confidence interval; OR, odds ratio.

### Logistic regression analysis to assess the association between alcohol consumption and the occurrence of eCLE in male and female participants, stratified by hiatal hernia

Next, we conducted a binary logistic regression analysis to assess the association in the occurrence of eCLE between alcohol intake and status of hiatal hernia in male participants (Table 4). Alcohol intake (≥20 g/day) was significantly associated with the incidence of eCLE in male participants without hiatal hernia (0 vs. ≥20 g/day; OR, 1.98; 95% CI, 1.04–4.03; *P* = 0.04) but not in male participants with hiatal hernia (0 vs. ≥20 g/day; OR, 1.04; 95% CI, 0.60–1.78; *P* = 0.88). This phenomenon was not observed in female participants (Table 5). Furthermore, there was no significant association between the occurrence of eCLE and alcohol intake simply stratified by sex (S2 Table).

**Table 4:** Logistic regression analysis to assess the association between alcohol consumption and the occurrence of endoscopic columnar-lined esophagus, stratified by hiatal hernia in male participants.

				Endoscopic columnar-lined esophagus (Outcome variable†)	
		No. of	No. of	Univariable	Multivariable
		cases	eCLE	OR (95% CI)	OR (95% CI)*
Hiatal hernia (+)	Total	1732	83 (4.8%)		
Alcohol consumption	None	512	25 (4.9%)	1 (reference)	1 (reference)
	<20 g/day	534	24 (4.5%)	0.98 (0.57–1.67)	1.00 (0.56–1.79)
	≥20 g/day	686	34 (5.0%)	1.09 (0.61–1.94)	1.04 (0.60–1.78)
	*P* value for 0 vs. <20 g/day			0.95	0.99
	*P* value for 0 vs. >20 g/day			0.77	0.88
Hiatal hernia (-)	Total	3282	53 (1.6%)		
Alcohol consumption	None	882	12 (1.4%)	1 (reference)	1 (reference)
	< 20g/day	1205	8 (0.7%)	0.48 (0.19–1.18)	0.50 (0.19–1.22)
	≥ 20g/day	1195	33 (2.8%)	2.05 (1.09–4.13)	1.98 (1.04–4.03)
	*P* value for 0 vs. <20 g/day			0.11	0.13
	*P* value for 0 vs. >20 g/day			0.03	0.04

†Presence of endoscopic columnar-lined esophagus was defined by more than 10 mm length of columnar-lined esophagus on upper endoscopy.

* The odds ratio was adjusted for age, body mass index, current smoking, presence of heartburn or acid regurgitation, presence of erosive esophagitis, presence of NERD, and presence of atrophic gastritis.

BMI, body mass index; CI, confidence interval; NERD, non-erosive reflex disease; OR, odds ratio

**Table 5:** Logistic regression analysis to assess the association between alcohol consumption and the occurrence of endoscopic columnar-lined esophagus stratified by hiatal hernia status in female participants.

				Endoscopic columnar-lined esophagus (Outcome variable†)	
		No. of	No. of	Univariable	Multivariable
		cases	eCLE	OR (95% CI)	OR (95% CI)*
Hiatal hernia (+)	Total	575	16 (2.8%)		
Alcohol consumption	None	357	10 (2.8%)	1 (reference)	1 (reference)
	< 20g/day	170	3 (1.8%)	0.62 (0.14–2.07)	0.70 (0.15–2.39)
	≥ 20g/day	48	3 (6.3%)	2.31 (0.50–7.90)	2.32 (0.46–8.82)
	*P* value for 0 vs. <20g/day			0.46	0.59
	*P* value for 0 vs. >20g/day			0.25	0.27
Hiatal hernia (-)	Total	2442	22 (0.9%)		
Alcohol consumption	None	1472	17 (1.4%)	1 (reference)	1 (reference)
	< 20g/day	755	4 (0.5%)	0.46 (0.13–1.23)	0.47 (0.14–1.29)
	≥ 20g/day	215	1 (0.5%)	0.40 (0.02–1.96)	0.47 (0.03–2.37)
	*P* value for 0 vs. <20g/day			0.13	0.15
	*P* value for 0 vs. >20g/day			0.31	0.42

†Presence of endoscopic columnar-lined esophagus was defined by more than 10 mm length of columnar-lined esophagus on upper endoscopy.

* The odds ratio was adjusted for age, body mass index, current smoking, presence of heartburn or acid regurgitation, presence of erosive esophagitis, presence of NERD, and presence of atrophic gastritis.

BMI, body mass index; CI, confidence interval; NERD, non-erosive reflex disease; OR, odds ratio

In male participants, hiatal hernia (presence: OR, 2.79; 95% CI, 1.96–4.01; *P* < 0.0001), age (10-year increments: OR, 1.49; 95% CI, 1.25–1.77; *P* < 0.0001), and erosive esophagitis (presence: OR, 2.00; 95% CI, 1.38–2.87; *P* = 0.0002) were risk factors of eCLE in the multivariate logistic regression analysis. Meanwhile, in female participants, only hiatal hernia (presence: OR, 2.88; 95% CI, 1.45–5.56; *P* = 0.002) was the risk factor of eCLE in the multivariate logistic regression analysis (S3 Table).

## Discussion

In this large prospective, multicenter, cohort study, we found a significant association in the occurrence of eCLE between alcohol intake and status of hiatal hernia in male participants. Our data supported the hypothesis that the effect of alcohol consumption might be associated with hiatal hernia status and male sex. With the large number of cases, to the best of our knowledge, this is the first study to examine the association in the occurrence of eCLE among alcohol intake, status of hiatal hernia, and male sex.

Hiatal hernia, GERD (and GERD symptoms), male sex, smoking, and obesity have been reported as risk factors of BE in many previous reports–many of which were in Western countries [12, 15, 25–27]. One recent meta-analysis showed the prevalence and risk factors of BE in Asian countries [28]. In that report, the pooled prevalence of eCLE and histologically confirmed BE was 7.8% and 1.3%, respectively. Most histologically confirmed BEs were cases of SSBE. Reflux symptoms (pooled OR = 3.15, 95% CI = 1.61–6.17), male sex (pooled OR = 1.50, 95% CI = 1.11–2.03), hiatal hernia (pooled OR = 4.88, 95% CI = 2.93–8.13), and smoking (pooled OR = 1.26, 95% CI = 1.01–1.56) were associated with a significantly increased risk of BE. Despite current smoking not having significant association with eCLE, other factors were significantly or likely to be associated with eCLE in our study (Table 2). NERD and the absence of atrophic gastritis were the factors associated with reflux symptoms [29] [30]. These factors were not associated with the incidence of eCLE in this study.

In our study, the effect of alcohol intake on the association between the occurrence of eCLE and hiatal hernia was limited to male participants. Some previous reports suggested that the anti-inflammatory action of estrogen and esophageal epithelial resistance against reflux of gastric acid were associated with the occurrence of BE [17, 18]. In our study, alcohol intake (≥20 g/day) was more frequently observed in male than in female participants, which might be one of the reasons for the high frequency of eCLE in male participants.

The influence of alcohol intake on the incidence of BE has been inconsistent. Alcohol has been shown to reduce LES pressure or lead to a loss of normal regulation of LES function, which, in turn, can lead to increased GERD symptoms [31–33]. Alcohol consumption was reported to lead to a 1.2- to 2.9-fold increased risk of GERD [34, 35]. However, many reports showed a negative association between alcohol consumption and incidence of BE [10, 11]. We hypothesized a significant relationship between alcohol consumption and occurrence of BE in a specific condition. In our study, the effect of alcohol consumption might be limited to male participants without hiatal hernia. This can be attributed to the fact that hiatal hernia can lead to a loss of normal regulation of LES pressure and alcohol consumption does not affect LES pressure in patients with hiatal hernia. Interestingly, the J curve phenomenon was observed in most analyses. Mild alcohol consumption (<20 g/day) would reduce the risk for BE (Tables 3, 4 and 5), whereas a greater alcohol intake (≥20 g/day) might be associated with an increased risk of BE.

Socioeconomic status was also reported as a risk factor for BE [36]. In this study, we did not have access to this information. However, the socioeconomic status of the majority of our participants was likely middle-class because they could pay the relatively expensive health care costs themselves or with support from their employers.

The strength of this study is the use of a large amount of data from a prospective multicenter cohort study. The size and comprehensiveness of this database enabled us to examine the independent association in the occurrence of eCLE among alcohol consumption, male sex, and hiatal hernia, while adjusting for potential confounders. However, our study has some limitations. BE was not confirmed histologically. In Asia, most cases of BE are SSBEs, and it is sometimes difficult to perform multiple biopsies from this region. However, one report showed that the overall prevalence of histological BE in eCLE was 31.7% [28]. Histological confirmation should be included in a future study. Another limitation is the lack of information about alcohol consumption duration (including none) and alcohol type. Additional studies are necessary to clarify whether alcohol consumption or alcohol intake cessation has any additional or independent effect beyond that reported in this study. Thrift AP et al reported that wine was associated with a moderately reduced risk for BE [11]. Therefore, further examination, stratified by alcohol type, is necessary. Moreover, there were no esophageal adenocarcinoma cases associated with BE in this study. The UGID study is a 5-year follow-up study, and additional information will appear in the near future and clarify the effect of alcohol intake on the occurrence of esophageal adenocarcinoma.

In conclusion, the association of alcohol intake with the incidence of eCLE might be related to hiatal hernia status and male sex. To evaluate the effect of alcohol consumption on the incidence of BE, the condition of hiatal hernia and male sex should be considered.

## Supporting information

S1 Table Association between alcohol consumption and the occurrence of an endoscopic columnar-lined esophagus, stratified by hiatal hernia status. (DOCX)

S2 Table Logistic regression analysis to assess the association between alcohol consumption and the occurrence of an endoscopic columnar-lined esophagus, stratified by sex. (DOCX)

S3 Table Logistic regression analysis of risk factors for endoscopic columnar-lined esophagus, stratified by sex. (DOCX)

S1 File Upper gastrointestinal disease study questionnaire. (DOCX)
