# Supplementary material for: Influence of hiatal hernia and male sex on the relationship between alcohol intake and occurrence of Barrett’s esophagus
Source: PLoS One. 2018 Feb 15;13(2):e0192951. doi: 10.1371/journal.pone.0192951 (PMC5814023; doi:10.1371/journal.pone.0192951)
Supplement: S1 Table — (DOCX) [file pone.0192951.s001.docx]

**S1 Table. Association between alcohol consumption and occurrence of endoscopic columnar-lined esophagus stratified by hiatal hernia degree**

|  | |  |  |  |
| --- | --- | --- | --- | --- |
|  | | No. of | No. of |  |
|  | | cases | eCLE | *P* value^#^ |
| Hiatal hernia causing proximal dislocation of GEJ > 4 cm above the diaphragmatic indentation | | 290 | 25 (8.6%) |  |
| Alcohol consumption | None | 88 | 9 (10.2%) |  |
|  | <20 g/day | 89 | 8 (9.0%) |  |
|  | ≥20 g/day | 113 | 8 (7.1%) | 0.72 |
| Hiatal hernia causing proximal dislocation of the GEJ 2–4 cm above the diaphragmatic indentation | | 2017 | 74 (3.7%) |  |
| Alcohol consumption | None | 781 | 26 (3.3%) |  |
|  | <20 g/day | 615 | 19 (3.1%) |  |
|  | ≥20 g/day | 621 | 29 (4.7%) | 0.27 |
| Hiatal hernia (-) | | 5724 | 75 (1.3%) |  |
| Alcohol consumption | None | 2354 | 29 (1.0%) |  |
|  | <20 g/day | 1960 | 12 (0.6%) |  |
|  | ≥20 g/day | 1410 | 34 (2.4%) | <0.0001 |

Presence of endoscopic columnar-lined esophagus was defined as columnar-lined esophagus length ≥ 10 mm on upper endoscopy.

^#^*P* value was calculated by a two-sided Fisher’s exact test.
