# Supplementary material for: Influence of hiatal hernia and male sex on the relationship between alcohol intake and occurrence of Barrett’s esophagus
Source: PLoS One. 2018 Feb 15;13(2):e0192951. doi: 10.1371/journal.pone.0192951 (PMC5814023; doi:10.1371/journal.pone.0192951)
Supplement: S2 Table — (DOCX) [file pone.0192951.s002.docx]

**S2 Table. Logistic regression analysis to assess association between alcohol consumption and occurrence of endoscopic columnar-lined esophagus stratified by sex**

|  | |  |  | Endoscopic columnar-lined esophagus  (Outcome variable†) | |
| --- | --- | --- | --- | --- | --- |
|  | | No. of | No. of | Univariable | Multivariable |
|  | | cases | eCLE | OR (95% CI) | OR (95% CI)* |
| Male | Total | 5014 | 136 (2.7%) |  |  |
| Alcohol  consumption | None | 1394 | 37 (2.7%) | 1 (reference) | 1 (reference) |
|  | < 20g/day | 1739 | 32 (1.8%) | 0.69 (0.42-1.10) | 0.74 (0.45-1.19) |
|  | ≥ 20g/day | 1881 | 67 (3.6%) | 1.35 (0.91-2.05) | 1.25 (0.83-1.91) |
|  | *P* value for  0 vs. <20g/day |  |  | 0.12 | 0.21 |
|  | *P* value for  0 vs. >20g/day |  |  | 0.14 | 0.28 |
| Female | Total | 3017 | 38 (1.3%) |  |  |
| Alcohol  consumption | None | 1829 | 27 (1.5%) | 1 (reference) | 1 (reference) |
|  | < 20g/day | 925 | 7 (0.8%) | 0.51 (0.20-1.11) | 0.54 (0.22-1.19) |
|  | ≥ 20g/day | 263 | 4 (1.5%) | 1.03 (0.30-2.66) | 1.15 (0.33-3.04) |
|  | *P* value for  0 vs. <20g/day |  |  | 0.09 | 0.13 |
|  | *P* value for  0 vs. >20g/day |  |  | 0.96 | 0.81 |

†Presence of endoscopic columnar-lined esophagus was defined as columnar-lined esophagus length ≥ 10 mm on upper endoscopy.

*The odds ratio was adjusted for age, body mass index, current smoking, presence of heartburn or acid regurgitation, presence of erosive esophagitis, presence of hiatal hernia, presence of atrophic gastritis, and presence of NERD.

BMI, body mass index; CI, confidence interval; NERD, non-erosive reflex disease; OR, odds ratio
