# Supplementary material for: Influence of hiatal hernia and male sex on the relationship between alcohol intake and occurrence of Barrett’s esophagus
Source: PLoS One. 2018 Feb 15;13(2):e0192951. doi: 10.1371/journal.pone.0192951 (PMC5814023; doi:10.1371/journal.pone.0192951)
Supplement: S3 Table — (DOCX) [file pone.0192951.s003.docx]

S3 Table.. Logistic regression analysis of risk factors for endoscopic columnar-lined esophagus stratified by sex

| **Male (N = 5014)** |  |  |
| --- | --- | --- |
| Univariable analysis | OR (95% CI) | *P* value |
| Hiatal hernia (presence) | 3.06 (2.17-4.37) | <0.0001 |
| Erosive esophagitis (presence) | 2.40 (1.69-3.40) | <0.0001 |
| Age (10-year increments) | 1.41 (1.19-1.68) | <0.0001 |
| Heartburn or acid regurgitation (presence) | 1.63 (1.16-2.30) | 0.005 |
| Alcohol consumption (≥20 g/day) | 1.64 (1.16-2.31) | 0.005 |
| Multivariate analysis* |  |  |
| Hiatal hernia (presence) | 2.79 (1.96-4.01) | <0.0001 |
| Age (10-year increments) | 1.49 (1.25-1.77) | <0.0001 |
| Erosive esophagitis (presence) | 2.00 (1.38-2.87) | 0.0002 |
| Alcohol consumption (≥20 g/day) | 1.48 (1.05-2.10) | 0.03 |
| **Female (N = 3017)** |  |  |
| Univariate analysis | OR (95% CI) | *P* value |
| Hiatal hernia (presence) | 3.15 (1.62-6.00) | 0.001 |
| Erosive esophagitis (presence) | 2.91 (1.17-6.33) | 0.02 |
| Multivariate analysis* |  |  |
| Hiatal hernia (presence) | 2.88 (1.45-5.56) | 0.002 |

Presence of endoscopic columnar-lined esophagus was defined as a columnar-lined esophagus length ≥ 10 mm on upper endoscopy.

*The odds ratio was adjusted for age, body mass index, current smoking, alcohol consumption, presence of heartburn or acid regurgitation, presence of erosive esophagitis, presence of NERD, presence of hiatal hernia and presence of atrophic gastritis.

BMI, body mass index; CI, confidence interval; NERD, non-erosive reflex disease; OR, odds ratio
